# Effects of compound probiotics and aflatoxin-degradation enzyme on alleviating aflatoxin-induced cytotoxicity in chicken embryo primary intestinal epithelium, liver and kidney cells

**DOI:** 10.1186/s13568-021-01196-7

**Published:** 2021-03-01

**Authors:** Hong-Wei Guo, Juan Chang, Ping Wang, Qing-Qiang Yin, Chao-Qi Liu, Xiao-Xiang Xu, Xiao-Wei Dang, Xiao-Fei Hu, Quan-Liang Wang

**Affiliations:** 1 College of Animal Science and Technology, Henan Agricultural University, Zhengzhou, 450046 China; 2 Henan Delin Biological Product Co., Ltd, Xinxiang, 453000 China; 3 Henan Key Laboratory of Animal Immunology, Henan Academy of Agricultural Sciences, Zhengzhou, 450002 China; 4 Henan Guangan Biotechnological Co., Ltd., Zhengzhou, 450001 China

**Keywords:** Aflatoxin B_1_, Compound probiotics, Mycotoxin-degradation enzyme, Chicken embryo primary cells, Cell damage alleviation

## Abstract

Aflatoxin B_1_ (AFB_1_) is one of the most dangerous mycotoxins for humans and animals. This study aimed to investigate the effects of compound probiotics (CP), CP supernatant (CPS), AFB_1_-degradation enzyme (ADE) on chicken embryo primary intestinal epithelium, liver and kidney cell viabilities, and to determine the functions of CP + ADE (CPADE) or CPS + ADE (CPSADE) for alleviating cytotoxicity induced by AFB_1_. The results showed that AFB_1_ decreased cell viabilities in dose-dependent and time-dependent manners. The optimal AFB_1_ concentrations and reactive time for establishing cell damage models were 200 µg/L AFB_1_ and 12 h for intestinal epithelium cells, 40 µg/L and 12 h for liver and kidney cells. Cell viabilities reached 231.58% (*p* < 0.05) for intestinal epithelium cells with CP addition, 105.29% and 115.84% (*p* < 0.05) for kidney and liver cells with CPS additions. The further results showed that intestinal epithelium, liver and kidney cell viabilities were significantly decreased to 87.12%, 88.7% and 84.19% (*p* < 0.05) when the cells were exposed to AFB_1_; however, they were increased to 93.49% by CPADE addition, 102.33% and 94.71% by CPSADE additions (*p* < 0.05). The relative mRNA abundances of IL-6, IL-8, TNF-α, iNOS, NF-κB, NOD1 (except liver cell) and TLR2 in three kinds of primary cells were significantly down-regulated by CPADE or CPSADE addition, compared with single AFB_1_ group (*p* < 0.05), indicating that CPADE or CPSADE addition could alleviate cell cytotoxicity and inflammation induced by AFB_1_ exposure through suppressing the activations of NF-κB, iNOS, NOD1 and TLR2 pathways.

## Keypoints

- AFB_1_ decreased chicken embryo primary intestinal epithelium, liver and kidney cell viabilities in dose-dependent and time-dependent manners.
- CPADE or CPSADE was able to relieve cell damages exposed to AFB_1_.
- CPADE or CPSADE addition could alleviate cell cytotoxicity and inflammation induced by AFB_1_ through suppressing the activations of NF-κB, iNOS, NOD1 and TLR2 pathways.

## Introduction

Mycotoxins are toxigenic fungal secondary metabolites that mainly produced by *Aspergillus*, *Penicillium* and *Fusarium* to have great threat to human and animal health globally. The Food and Agriculture Organization (FAO) showed that approximately 25% of worldwide agricultural raw materials were contaminated with mycotoxins, leading to health problems and enormous economic losses (FAO 2013). So far, at least 400 kinds of mycotoxins such as aflatoxins, zearalenone, deoxynivalenol, fumonisin, patulin, T-2 toxin and ochratoxins have been identified (Cimbalo et al. 2020). There are more than 20 types of aflatoxins including aflatoxin B_1_ (AFB_1_), B_2_, G_1_, G_2_ and M_1_, among them AFB_1_ is the most toxic mycotoxin with high frequency of contamination in various cereals such as nuts, corn and rice (Negash 2018). AFB_1_ is able to cause poor feed efficacy, hepatotoxic, carcinogenic, teratogenic, immunosuppressive and other devastating effects on humans and animals (Meissonnier et al. 2008; Trebak et al. 2015; Zhang et al. 2016). Therefore, it is classified as the category one carcinogen by the International Agency for Research on Cancer (IARC 2012).

Poultry is more sensitive to AFB_1_ than the other kinds of animals. AFB_1_ residues in poultry body will cause potential health hazard for humans and itself (Peng et al. 2014). It is known that moldy food contains large amounts of AFB_1_, especially in moldy peanuts and cereals. In poultry farming, AFB_1_ can severely affect the immune system to cause immunosuppression (Liu et al. 2016). AFB_1_ can also cause apoptosis, gross and histopathological lesions in different organs, especially in liver, kidney, muscles and bursa of Fabricius (Chen et al. 2014; Peng et al. 2014). It was reported that AFB_1_ intoxication could increase mortality, liver and kidney pathology, and decrease bodyweight and feed intake for broilers (Saleemi et al. 2019). Therefore, it is necessary to develop effective detoxification strategies to increase AFB_1_ degradation and alleviate AFB_1_-induced inflammatory and immunosuppression in chickens.

Up to date, several strategies have been reported to alleviate AFB_1_ toxicity including physical, chemical and biological methods. The physical detoxification methods (absorption, heating and irradiation) and chemical detoxification methods (ammonization, solvent extraction and oxidation) have many defects such as nutritional losses, expensive equipment requirement and low efficiency (Gregorio et al. 2014; Arzandeh and Jinap 2015; Zhu et al. 2016). It was found that the biological method was more effective to degrade mycotoxins than other ones (Das et al. 2014; Melvin et al. 2014; Fernández et al. 2015). Many species of microbes such as bacteria, molds and yeasts have demonstrated the capability to alleviate AFB_1_ toxicity, due to their metabolic transformation or adsorption ability for AFB_1_. It was reported that addition of lactic acid bacteria and *S. cerevisiae* to AFB_1_-contaminated diet could reduce AFB_1_ residues and prevent degenerative changes in the liver and kidney of broilers (Śliżewska et al. 2019). *Aspergillus oryzae* has been reported to be able to degrade AFB_1_ (Alberts et al. 2009). The other reports showed that the cooperation of compound probiotics (CP) and AFB_1_-degradation enzyme (ADE) could degrade AFB_1_ effectively (Zuo et al. 2013; Huang et al. 2019).

It was reported that liver and kidney were the primary target organs attacked by AFB_1_ (Gholami-Ahangaran et al. 2016; Pérez-Acosta et al. 2016). In addition, the small intestine is the physical barrier which usually first contacts with and absorbs AFB_1_, as a result intestinal heath is seriously influenced by AFB_1_ (Pinton and Oswald 2014). However, the optimal strategies for alleviating the negative effects of AFB_1_ on intestine, liver and kidney cells of chickens have not been reported. Therefore, small intestine, liver and kidney cells of chickens were selected in this study to investigate the toxic effects of AFB_1_ on chicken embryo primary cells, and explore the efficacy of CPADE or CPSADE for alleviating AFB_1_-induced cytotoxicity and inflammatory of chickens.

## Materials and methods

### Chemicals and AFB_1_ preparation

Phosphate-buffered saline (PBS), 0.25% pancreatin with ethylenediaminetetraacetic acid (EDTA), collagenase (C8140, 246 U/mg), neutral protease (D6430, 0.5 U/mg), penicillin–streptomycin and thiazolyl blue tetrazolium bromide (MTT) were purchased from Beijing Solarbio Biotechnology Co., Ltd. Beijing, China. Collagenase and protease were dissolved in PBS to make 3000 U/mL and 0.5 U/mL, respectively. Percoll separation solution was diluted with PBS to 50%. Dulbecco’s Modified Eagle Medium/Nutrient Mixture F-12 (DMEM/F12 at 1/1), M199 medium and fetal bovine serum (FBS) were purchased from Biological Industries (Kibbutz Beit-Haemek, Israel). Aflatoxin B_1_ was purchased from Sigma-Aldrich (St. Louis, MO, U.S.), dissolved in 50% methanol to make 8 μg/mL AFB_1_ concentration as the stock solution, filtered with 0.22 μm membrane high-flow filter (Sartorius Stedim Biotech Gmbh, Goettingen, Germany), and stored at 4 °C for the following experiment.

### Probiotics and AFB_1_-degrading enzyme preparation

Based on the previous research in our laboratory, four species of microorganisms with high AFB_1_-degrading abilities including *Bacillus subtilis* (*B. subtilis*, CGMCC1.0504), *Enterococcus faecalis* (*E. faecalis*, CGMCC1.2135), *Candida utilis* (C*. utilis*, CGMCC2.0615) and *Lactobacillus casein* (*L. casein*, CGMCC1.2884) were selected, which were purchased from China General Microbiological Culture Collection Center (CGMCC), Beijing, China. The microbes were incubated to more than 1.0 × 10^9^ CFU/mL according to the published protocols (Huang et al. 2018). After centrifugation at 4 °C and 12,000×*g* for 10 min, the microbes and supernatants were collected, respectively. The supernatants were filtered through 0.22 µm membrane to remove the microbes, and then diluted to the final concentrations required by experiment design with cell media for subsequent experiments. The microbes were also adjusted to the different concentrations with cell media. Based on the previous results obtained with response surface regression design in our laboratory in vitro, the optimal final counts of *B. subtilis*, *L. casein*, *E. faecalis* and *C. utilis* for AFB_1_-degradation were 1.0 × 10^5^, 1.0 × 10^5^, 1.0 × 10^7^ and 1.0 × 10^5^ CFU/mL to make the basal compound probiotics (CP). In order to measure the effects of different CP concentrations on cell viability or alleviating AFB_1_-induced cytotoxicity, the final counts of *B. subtilis*, *L. casein*, *E. faecalis* and *C. utilis* in CP were further designed as 1.0 × 10^2^, 1.0 × 10^2^, 1.0 × 10^4^ and 1.0 × 10^2^ CFU/mL to make CP1; 1.0 × 10^3^, 1.0 × 10^3^, 1.0 × 10^5^ and 1.0 × 10^3^ CFU/mL to make CP2; 1.0 × 10^4^, 1.0 × 10^4^, 1.0 × 10^6^ and 1.0 × 10^4^ CFU/mL to make CP3; 1.0 × 10^5^, 1.0 × 10^5^, 1.0 × 10^7^ and 1.0 × 10^5^ CFU/mL to make CP4; 1.0 × 10^6^, 1.0 × 10^6^, 1.0 × 10^8^ and 1.0 × 10^6^ CFU/mL to make CP5, respectively. Their corresponding supernatants were combined together to make CPS1, CPS2, CPS3, CPS4 and CPS5.

The AFB_1_-degradating enzyme was extracted from solid-state fermentation of *Aspergillus oryzae* (*A. oryzae*, CGMCC3.4437) according to the previous protocol (Huang et al. 2019). The crude enzyme solution of 10% AFB_1_-degrading enzyme was diluted with cell medium and stored at 4 °C for further use. The AFB_1_-degrading enzyme activity in 10% crude enzyme solution was determined to be 51 U/mL according to the previous protocol (Gao et al. 2011).

### Primary chicken embryo intestinal epithelium, liver and kidney cell preparation

The 14-day-old fertilized chicken eggs were purchased from Kaifeng Breeding Chicken Co., Ltd. Kaifeng, China, which were cleaned by 75% alcohol, placed in a vertical-flow clean bench ultra-clean, and handled with ultraviolet irradiation for 20 min. The air chamber of embryo was carefully broken with the tweezers, the chicken embryo was taken out and quickly decapitated, followed by taking out small intestine, liver and kidney tissues, and rinsed in PBS containing 1% penicillin (10,000 U/mL)-streptomycin (10 mg/mL) (Beijing Solarbio Biotechnology Co., Ltd. Beijing, China).

The mesentery of small intestine was carefully exfoliated in PBS solution, cut into 1 mm size, put into 5 mL centrifuge tube, and washed with PBS until the supernatant was clear. After removing the washing solution, 1 mL 0.25% pancreatin was added to digest the tissues at 37 °C for 10 min with shaking once every 2 min. The tissues were centrifuged at 1000 r/min for 5 min to remove supernatant, and then 2 mL DMEM/F12 medium supplemented with 10% FBS and 1% penicillin–streptomycin were added. The filtrate was collected using 200-mesh sieve, and the cells were cultured in a 5% CO_2_ incubator at 37 °C for 2 h. The supernatant was removed after centrifuged with 1000 r/min for 10 min, the cells were adjusted to 5.0 × 10^5^ cells/mL with DMEM/F12 supplemented with 2.5% FBS and 1% penicillin–streptomycin. 0.2 mL or 2 mL cells were put in a 96-well or 12-well culture plate, and cultured at 37 °C in a 5% CO_2_ incubator. The incubating cell medium was replaced every 2 days.

Liver cells were prepared as above and modified as following: 1 mL collagen protease and 1 mL neutral protease were added to digest the tissues at 37 °C for 30 min with shaking once every 3 min. Then 2 mL M199 medium supplemented with 10% FBS and 1% penicillin–streptomycin were added. After shaking up and down, the filtrates were collected with a 200-mesh sieve, and then centrifuged with 1000 r/min for 10 min to remove the supernatant. 1.5 mL M199 medium supplemented with 10% FBS and 1% penicillin–streptomycin were added to the centrifuge tube, and then 3 mL 50% percoll separation solution were added and mixed well, centrifuged for 15 min at 3000 r/min. After centrifugation, the upper layer was removed, and the middle layer was taken out and put into a new centrifuge tube, then equivalent volume M199 medium was added to the new centrifuge tube, centrifuged for 10 min at 1000 r/min. At last the liver cells were resuspended with M199 medium supplemented with 10% FBS and 1% penicillin–streptomycin, adjusted and cultured as above. Kidney cells were prepared with the same protocol as liver cells, modified by using DMEM/F12 medium to replace M199 medium.

### Cell viability assay and experimental design

Three kinds of primary cells were seeded into 96-well plates. Cell viability was measured by MTT assay every 2 days (Fotakis and Timbrell 2005). The growth curves of three kinds of cells were plotted with time as the abscissa and absorbance value as the ordinate. The following experiments were carried out in the logarithmic phase of cells. The experimental designs were as follows:

1. Effect of different AFB_1_ concentrations on cell damage: three kinds of cells were seeded into 96-well plates with a density of 5.0 × 10^5^ cells/mL, cultured to their logarithmic phases, followed by removing the culture medium and washing twice with PBS, and subsequently incubated with different concentrations of AFB_1_ for 6, 12, 24 and 48 h, respectively. AFB_1_ concentrations were 0, 40, 80, 120, 160 and 200 µg/L for intestinal epithelium cells; 0, 10, 20, 40 and 80 µg/L for the liver and kidney cells. AFB_1_ was diluted with the corresponding cell media without serum and antibiotics.
2. Effect of CP or CPS on cell viability: the cells were prepared as above. CP and CPS were diluted with the corresponding cell media without serum and antibiotics. The cells were incubated with the different concentrations of CP or CPS for 12, 24 and 48 h, respectively.
3. Effect of ADE on cell viability: ADE was diluted with the cell medium without serum and antibiotics to make the final concentrations at 0, 0.0001%, 0.001%, 0.01%, 0.1% and 1%, which was incubated with cells for 6, 12, 24 and 48 h, respectively.
4. The functions of CPADE and CPSADE for alleviating cytotoxicity: The cell culture was 12 h. The detail design was listed in Table 1. The previous report in our laboratory showed that CPADE and CPSADE were more effective than CP, CPS and ADE for degrading AFB_1_ (Huang et al. 2018); therefore, CP, CPS and ADE were not considered for alleviating cytotoxicity induced by AFB_1_ in this study.

**Table 1:** The experimental designs for CPADE or CPSADE to alleviate primary cell damages induced by AFB_1_

Primary cells	Control	AFB_1_ (µg/L)	CPADE or CPSADE	CPADE or CPSADE + AFB_1_
Intestinal epithelium cells	DMEM/F12	200	CP2 + 0.001%ADE	CP2 + 0.001%ADE + 200 µg/L AFB_1_
Liver cells	M199	40	CPS4 + 0.001%ADE	CPS4 + 0.001%ADE + 40 µg/L AFB_1_
Kidney cells	DMEM/F12	40	CPS3 + 0.001%ADE	CPS3 + 0.001%ADE + 40 µg/L AFB_1_

*CP* compound probiotics, *CPS* cell-free compound probiotics supernatant, *CPADE* compound probiotics + AFB_1_-degradation enzyme, *CPSADE* cell-free compound probiotics supernatant + AFB_1_-degradation enzyme

At the end of above cell incubations, each well was added with 10 µL 5 mg/mL MTT and incubated for 4 h. Then the cell supernatants were removed and 150 µL DMSO was added to each well. Thereafter, the plates were shaken for 10 min at room temperature. The absorbances (A) were determined at 490 nm wavelength with a reference wavelength of 630 nm by an ELx 800 microplate reader (BIO-TEK Instruments Inc., Winooski, VT, USA). The cell viability (%) = (A_490nm_ − A_630nm_ value in the experimental groups)/(A_490nm_ − A_630nm_ in the control groups) × 100%.

### Reverse transcription PCR and quantitative real-time PCR

The primary intestinal epithelium, liver and kidney cells were seeded with a density of 5.0 × 10^5^ cells/mL in 12-well culture plates and allowed to adhere for 24 h, respectively. After four treatments (Control, AFB_1_, CPADE or CPSADE, CPADE or CPASDE + AFB_1_) for three kinds of primary cells for 12 h respectively, total RNA was extracted using Trizol (Invitrogen, Carlsbad, CA, USA) according to the standard manufacturer’s instructions, and then dissolved in 50 µL RNase-free water, stored at − 80 °C. The quality and concentration of RNA samples were measured by NanoDrop ND-1000 Spectrophotometer (Nano-Drop Technologies, Wilmington, DE, U.S.). Approximately 1 µg total RNA from each sample was reversely transcribed into cDNA by TB GREEN kit (TaKaRa, Dalian, China). Quantitative RT-PCR was performed with CFX Connect™ Real-Time PCR Detection System (Bio-Rad, Hercules, CA, USA). All the primers used in this study were listed in Table 2. The β-actin was used as a house-keeping gene, and the relative gene abundances in chicken embryo primary intestinal epithelium, liver and kidney cells were analyzed using the 2^−ΔΔCT^ method (Livak and Schmittgen 2001).

**Table 2:** Primer sequences of some genes for quantitative RT-PCR

Gene	Accession number	Primer sequence (5′–3′)
β-actin	LO8165	F: GAGAAATTGTGCGTGACATCA
		R: CCTGAACCTCTCATTGCCA
IL-6	AJ309540	F: CAAGGTGACGGAGGAGGAC
		R: TGGCGAGGAGGGATTTCT
IL-8	AJ009800	F: ATGAACGGCAAGCTTGGAGCTG
		R: TCCAAGCACACCTCTCTTCCATCC
iNOS	U46504	F: CAGCTGATTGGGTGTGGAT
		R: TTTCTTTGGCCTACGGGTC
NF-κBp65	NM_205129	F: GTGTGAAGAAACGGGAACTG
		R: GGCACGGTTGTCATAGATGG
TNF-α	NM_204267	F: GAGCGTTGACTTGGCTGTC
		R: AAGCAACAACCAGCTATGCAC
NOD1	JX465487	F: AGCACTGTCCATCCTCTGTCC
		R: TGAGGGTTGGTAAAGGTCTGCT
TLR2	NM_001161650	F: GGGGCTCAGGCAAAATC
		R: AGCAGGGTTCTCAGGTTCACA

### Statistical analysis

All experimental data were presented as means ± standard deviations. The data were analyzed using one-way analysis of variance (ANOVA) by the Duncan method with SPSS 20.0 software (Sishu Software, Shanghai Co., Ltd. Shanghai, China). All graphs were generated using GraphPad Prism 8. Differences were considered as statistically significance at *p* < 0.05.

## Results

### The growth curves of primary intestinal epithelium, liver and kidney cells of chicken embryo

**Fig. 1:**
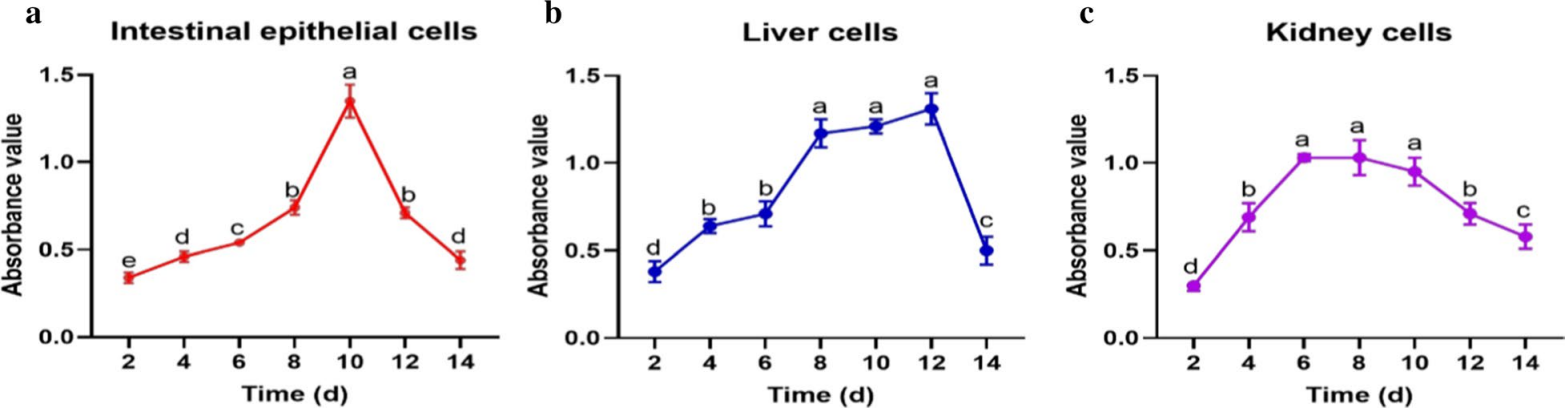
The growth curves of primary intestinal epithelium, liver and kidney cells (n = 8). The different lowercase letters indicate significant difference from each other (*p* < 0.05), while the same lowercase letters indicate insignificant difference from each other (*p* > 0.05)

Figure 1 demonstrated that the logarithmic growth phases of intestinal epithelium, liver and kidney cells appeared during the incubation periods of 8–12, 6–12 and 6–12 days, and reached the logarithmic peak on the 10th, 12th and 6th day, respectively (*p* < 0.05).

### Effects of AFB_1_ on the viabilities of primary intestinal epithelium, liver and kidney cells

Table 3 showed that AFB_1_ decreased cell viability in dose-dependent and time-dependent manners. The higher AFB_1_ concentrations and longer incubation time caused more serious damages for three kinds of cells. AFB_1_ had insignificant effect on intestinal epithelium cell viability when its concentration was below 80 μg/L within 48 h incubation (*p* > 0.05); however, it was significantly influenced when AFB_1_ concentration were more than 80 μg/L (*p* < 0.05), especially under the condition that the incubation time was 48 h. Liver and kidney cells of chicken embryo were more sensitive to AFB_1_ than intestinal epithelium cells. They were significantly influenced by 80 μg/L AFB_1_ within 6 h incubation, 40 μg/L AFB_1_ within 12 h incubation, 20 μg/L AFB_1_ within 24 h incubation, 10 μg/L AFB_1_ within 48 h incubation (*p* < 0.05), compared with the control group. After considering the above results, AFB_1_ concentrations and reaction time were confirmed as 200 μg/L and 12 h for intestinal epithelium cells, 40 μg/L AFB_1_ and 12 h for liver and kidney cells in the subsequent experiments.

**Table 3:** Effects of different AFB_1_ concentrations and incubation time on primary cell viability (%)

Time (h)	AFB_1_ concentrations (μg/L)					
	0	40	80	120	160	200
Intestinal epithelium cells						
6	100.00 ± 2.94^a^	105.88 ± 5.88^a^	102.94 ± 8.82^a^	91.18 ± 8.82^ab^	94.12 ± 5.88^ab^	88.24 ± 8.82^b^
12	100.00 ± 0.29^a^	100.00 ± 11.43^a^	102.86 ± 17.14^a^	97.14 ± 8.57^ab^	100.00 ± 8.57^a^	85.71 ± 7.14^b^
24	100.00 ± 14.63^a^	102.44 ± 2.44^a^	100.00 ± 2.44^a^	100.00 ± 2.44^a^	97.56 ± 4.88^a^	85.37 ± 7.32^b^
48	100.00 ± 5.13^a^	100.00 ± 5.13^a^	94.87 ± 7.69^ab^	82.01 ± 5.88^b^	83.66 ± 4.92^b^	48.72 ± 5.13^c^

	0	10	20	40	80	
Liver cells						
6	100.00 ± 4.88^ab^	109.76 ± 4.88^a^	112.2 ± 4.88^a^	100.00 ± 7.32^ab^	90.24 ± 4.88^b^	
12	100.00 ± 9.52^a^	109.52 ± 9.52^a^	104.76 ± 7.14^a^	80.95 ± 7.14^b^	85.71 ± 4.76^b^	
24	100.00 ± 2.13^a^	89.66 ± 5.31^b^	79.12 ± 4.54^c^	74.17 ± 3.68^c^	73.68 ± 3.29^c^	
48	100.00 ± 0.23^a^	68.18 ± 4.55^b^	72.73 ± 9.09^b^	79.55 ± 4.55^b^	75.00 ± 4.55^b^	
Kidney cells						
6	100.00 ± 5.56^a^	103.70 ± 3.70^a^	101.85 ± 5.56^a^	94.44 ± 5.56^a^	85.19 ± 3.70^b^	
12	100.00 ± 3.51^a^	96.49 ± 3.51^ab^	96.49 ± 5.26^ab^	89.47 ± 3.51^b^	71.53 ± 3.61^c^	
24	100.00 ± 4.69^a^	95.31 ± 4.69^ab^	87.50 ± 6.25^b^	81.25 ± 1.56^b^	56.25 ± 3.13^c^	
48	100.00 ± 6.15^a^	84.62 ± 3.08^b^	81.54 ± 1.54^b^	72.31 ± 3.08^c^	47.69 ± 4.62^d^	

Data were expressed as mean ± SD (n = 8). The different lowercase letters in the same row indicate significant difference from each other (*p* < 0.05), while the same lowercase letters in the same row indicate insignificant difference from each other (*p* > 0.05)

### Effects of CP or CPS on the viabilities of three kinds of primary cells

Table 4 showed that different concentrations of CP and CPS had different effects on three kinds of cell viabilities. The relative cell viabilities reached 231.58%, 163.33% and 138.32% (*p* < 0.05) for intestinal epithelium, liver and kidney cells at CP2 levels for 12 h incubation, respectively; which reached 136.13% and 115.84% (*p* < 0.05) at CPS4 levels after 12 h incubation for intestinal epithelium and liver cells, 105.29% (*p* < 0.05) at CPS3 levels after 12 h incubation for kidney cells. According to the above results, the optimal incubation time was selected as 12 h in the subsequent experiment. In general, the liver and kidney cells can’t directly contact with microbes; therefore, CPS was selected in the subsequent experiments for liver and kidney cell incubations.

**Table 4:** Effects of different CP or CPS concentrations and incubation time on primary cell viabilities (%)

	Time (h)	CP1 or CPS1	CP2 or CPS2	CP3 or CPS3	CP4 or CPS4	CP5 or CPS5
Intestinal epithelium cells						
CP	12	198.25 ± 10.53^b^	231.58 ± 5.26^c^	157.89 ± 5.26^a^	145.61 ± 7.02^a^	207.02 ± 3.51^b^
	24	130.23 ± 9.30^a^	120.93 ± 11.16^ab^	90.70 ± 8.10^d^	109.3 ± 7.67^c^	109.30 ± 6.60^bc^
	48	84.78 ± 8.70^a^	80.43 ± 4.35^a^	60.87 ± 3.04^b^	47.83 ± 4.35^c^	50.00 ± 3.52^c^
CPS	12	116.85 ± 5.01^bc^	113.88 ± 4.87^c^	105.39 ± 1.52^d^	136.13 ± 1.59 ^a^	122.24 ± 4.24 ^b^
	24	104.00 ± 3.50^d^	132.00 ± 11.4^c^	157.00 ± 2.80^a^	116.00 ± 3.70^b^	113.92 ± 5.80^b^
	48	103.00 ± 3.27^a^	102.00 ± 4.87^a^	104.00 ± 3.89^a^	99.00 ± 2.91^a^	104.00 ± 5.17^a^
Liver cells						
CP	12	141.67 ± 0.08^b^	163.33 ± 0.6^a^	130.00 ± 0.33^b^	110.00 ± 2.60^c^	103.33 ± 1.70^c^
	24	127.50 ± 0.10^a^	102.50 ± 1.25^c^	87.50 ± 1.20^e^	95.00 ± 1.60 ^d^	112.50 ± 1.13^b^
	48	68.09 ± 0.40^b^	59.57 ± 1.50^c^	59.57 ± 2.67^c^	78.72 ± 0.88^a^	80.85 ± 1.29 ^a^
CPS	12	99.87 ± 1.89^b^	99.76 ± 0.88^b^	102.41 ± 1.57^b^	115.84 ± 3.74^a^	114.07 ± 0.72^a^
	24	100.10 ± 1.26^b^	102.34 ± 1.26^b^	101.79 ± 2.19^b^	117.25 ± 1.99^a^	114.96 ± 6.46^a^
	48	99.12 ± 0.76^b^	97.37 ± 1.89^b^	96.01 ± 2.93^b^	102.09 ± 0.94^a^	102.94 ± 2.24^a^
Kidney cells						
CP	12	124.30 ± 4.67^b^	138.32 ± 1.87^a^	106.54 ± 1.87^c^	123.36 ± 10.28^b^	118.69 ± 4.67^b^
	24	120.00 ± 7.62 ^B^	130.05 ± 2.86^a^	72.38 ± 21.90^c^	53.33 ± 4.76^d^	56.19 ± 6.67^d^
	48	67.24 ± 3.45^a^	51.72 ± 1.72^b^	54.31 ± 19.83^b^	29.31 ± 8.62^c^	33.62 ± 4.31^c^
CPS	12	101.37 ± 1.18^b^	99.50 ± 2.26^b^	105.29 ± 1.34^a^	97.56 ± 3.67^b^	89.25 ± 1.28^c^
	24	100.58 ± 2.12^b^	102.25 ± 2.14^b^	111.30 ± 0.94^a^	100.41 ± 2.97^b^	77.78 ± 2.07^c^
	48	100.28 ± 1.33 ^B^	103.65 ± 2.43^b^	106.72 ± 5.81^a^	90.00 ± 2.05^c^	48.28 ± 2.66^d^

Data were expressed as mean ± SD (n = 8). The different lowercase letters in the same row indicate significant difference from each other (*p* < 0.05), while the same lowercase letters in the same row indicate insignificant difference from each other (*p* > 0.05). CP: compound probiotics (for intestinal epithelium cell incubation); CPS: cell-free compound probiotics supernatant (for liver and kidney cell incubation). The final counts of *B. subtilis*, *L. casein*, *E. faecalis* and *C. utilis* in CP were designed as 1.0 × 10^2^, 1.0 × 10^2^, 1.0 × 10^4^ and 1.0 × 10^2^ CFU/mL to make CP1; 1.0 × 10^3^, 1.0 × 10^3^, 1.0 × 10^5^ and 1.0 × 10^3^ CFU/mL to make CP2; 1.0 × 10^4^, 1.0 × 10^4^, 1.0 × 10^6^ and 1.0 × 10^4^ CFU/mL to make CP3; 1.0 × 10^5^, 1.0 × 10^5^, 1.0 × 10^7^ and 1.0 × 10^5^ CFU/mL to make CP4; 1.0 × 10^6^, 1.0 × 10^6^, 1.0 × 10^8^ and 1.0 × 10^6^ CFU/mL to make CP5, respectively. Their corresponding supernatants were combined together to make CPS1, CPS2, CPS3, CPS4 and CPS5

### Effects of ADE on viability of three kinds of primary cells

**Fig. 2:**
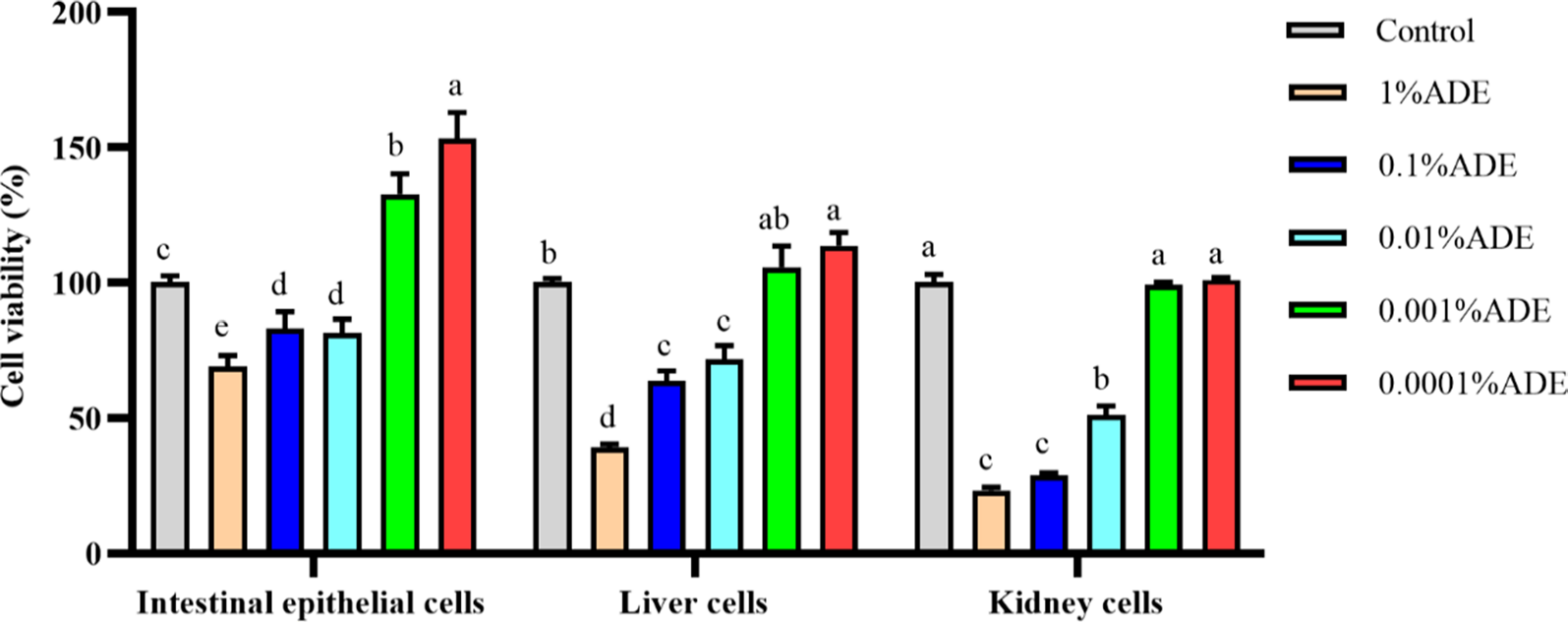
Effects of ADE on viabilities of primary intestinal epithelium, liver and kidney cells (n = 8). The different letters on each bar indicate significant difference from each other (*p* < 0.05), while the same letters on each bar indicate insignificant difference from each other (*p* > 0.05). *ADE* AFB_1_-degradation enzyme

Figure 2 showed that the relative viabilities of three kinds of cells were significantly decreased (*p* < 0.05) when ADE concentrations were between 0.01 and 1%; however, the cell viabilities were significantly increased when ADE concentrations were between 0.001 and 0.0001% (*p* < 0.05). Therefore, the optimal ADE content was selected as 0.001% in the subsequent experiment.

### Effects of CPADE or CPSADE on alleviating viabilities of three primary cells induced by AFB_1_

**Fig. 3:**
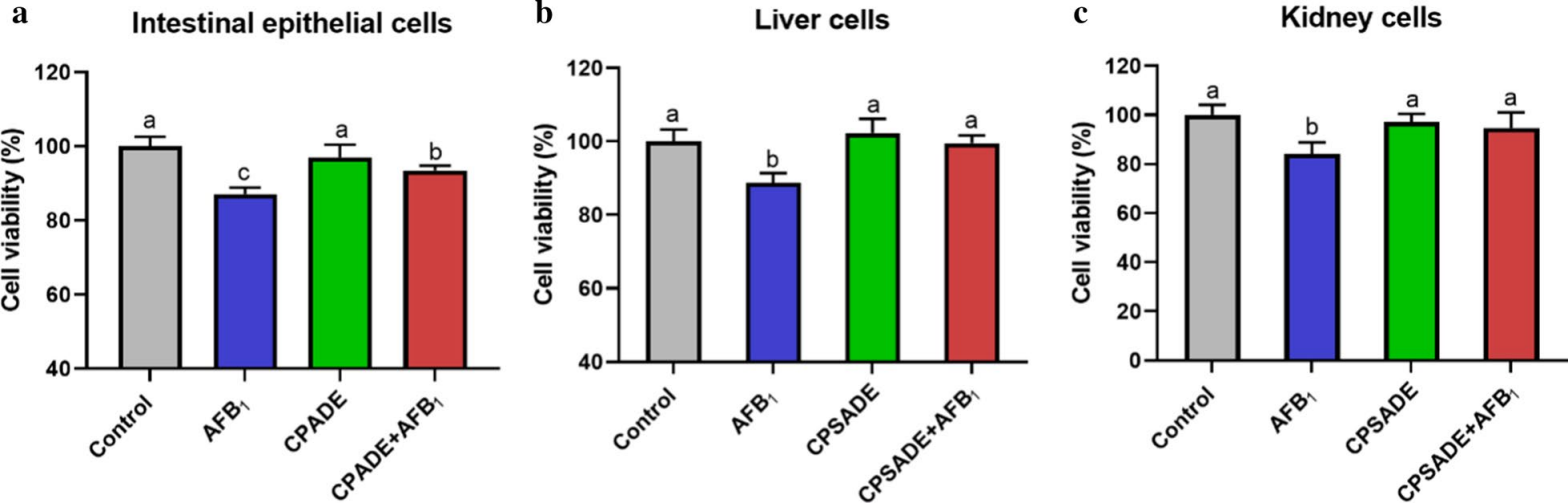
Effects of CPADE or CPSADE on alleviating viabilities of primary intestinal epithelium (**a**), liver (**b**) and kidney (**c**) cells induced by AFB_1_ after 12 h reaction (n = 8). The different letters on each bar indicate significant difference from each other (*p* < 0.05), while the same letters on each bar indicate insignificant difference from each other (*p* > 0.05). Control: DMEM/F12 or M199; AFB_1_: 200 μg/L for primary intestinal epithelium cells, 40 μg/L for liver and kidney cells; CPADE: compound probiotics + AFB_1_-degradation enzyme; CPSADE: cell-free compound probiotics supernatant + AFB_1_-degradation enzyme

Figure 3 showed that the relative viabilities of intestinal epithelium, liver and kidney cells induced by AFB_1_ were significantly decreased to 87.12%, 88.7% and 84.19% (*p* < 0.05), whereas CPADE or CPSADE addition significantly increased the cell viabilities to 93.49%, 102.33% and 94.71% (*p* < 0.05), respectively.

### Effects of CPDE or CPSADE on mRNA abundances of some genes related with cytokines and signal pathways in the three kinds of primary cells induced by AFB_1_

**Fig. 4:**
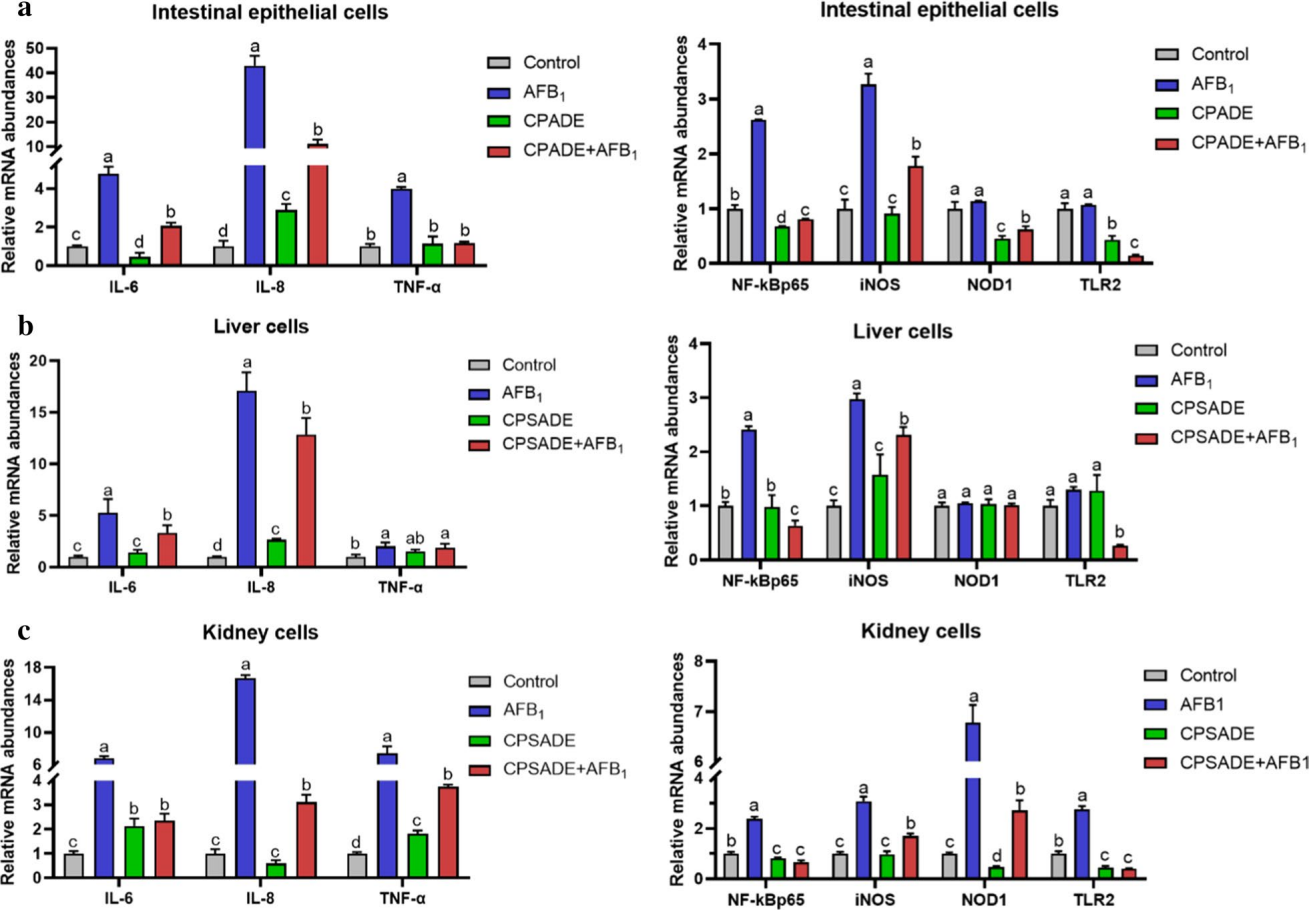
Effects of CPADE or CRSADE addition on mRNA abundances of some genes in primary intestinal epithelium (**a**), liver (**b**) and kidney (**c**) cells induced by AFB_1_ (n = 5). The different letters on each bar indicate significant difference from each other (*p* < 0.05), while the same letters on each bar indicate insignificant difference from each other (*p* > 0.05). Control: DMEM/F12 or M199; AFB_1_: 200 μg/L for primary intestinal epithelium cells, 40 μg/L for primary liver and kidney cells; CPADE: compound probiotics + AFB_1_-degradation enzyme; CPSADE: cell-free compound probiotics supernatant + AFB_1_-degradation enzyme

Figure 4 indicated that AFB_1_ exposures during intestinal epithelium, liver and kidney cell incubations could up-regulate the mRNA abundances of some genes such as IL-6, IL-8, TNF-α (except for liver), NF-κBp65, iNOS, NOD1 (except for liver) and TLR2 (*p* < 0.05); however, CPADE or CPSADE addition could retrieve the above results. It could be concluded that CPADE or CPSADE addition was able to alleviate cell inflammation induced by AFB_1_ through positively regulating some signal pathways.

## Discussions

Aflatoxins are the ubiquitous dietary contaminants all over the world, which lead to low feed intake, low efficiency and substantial economic losses (Tedesco et al. 2004). Aflatoxin B_1_ is frequently detected in cereals, feedstuffs and diets to cause liver damage and immune inhibition of domestic animals (Kraieski et al. 2016; Yuan et al. 2016). AFB_1_ residues in domestic animal products will be harmful to human and public health. Liver is the main target organ of AFB_1_, but AFB_1_ is also detected in kidney and intestinal tract of animals. Therefore, it is necessary to find an effective and safe method to alleviate AFB_1_ for animal and human. Nowadays, probiotics have been widely used to degrade mycotoxins. It was reported that *Bacillus subtilis* could germinate in intestinal tract, and reduce AFB_1_ absorption and residues in the internal organs of broilers (Salem et al. 2018). The compound probiotics of *B. subtilis*, *L. casei* and *C. utilis* were reported to increase production performance, alleviate histological lesions, degrade mycotoxins and decrease mycotoxin residues in broilers (Chang et al. 2020). In order to increase the efficiency of alleviating AFB_1_-induced cell damage, the compound probiotics was combined with AFB_1_-degrading enzyme in this study.

This result showed that the viabilities of three kinds of primary cells were decreased with increasing AFB_1_ concentrations and incubation time, suggesting that both of them are the main factors for determining the extent of AFB_1_ toxicity. In general, liver and kidney cells are more sensitive to AFB_1_ than intestinal cells, which may be related to the different responses from the different cell types and organs (Zain 2011). AFB_1_ can be metabolized to high reactive metabolites by cytochrome P450 enzyme system in liver cells, resulting in formation of AFB_1_-DNA adducts to cause carcinogenesis and mutations (Valeria et al. 2020; Owumi et al. 2020). The kidney cells can be directly damaged by AFB_1_ through increasing cell apoptosis and death (Li et al. 2019). For the intestinal epithelium cells, AFB_1_ damage was mainly presented from barrier function loss and inflammatory response (Hernández-Ramírez et al. 2019). Because intestinal epithelium cells usually contact with AFB_1_ directly, the long-term adaptation makes them be insensitive to AFB_1_ than liver and kidney cells. The addition of compound probiotics and mycotoxin-degrading enzyme could contribute to cell proliferations and alleviate the toxicity induced by AFB_1_, which might be from mycotoxin biodegradation (Huang et al. 2018). It was found that the different concentrations of CP or CPS at different reaction time had different effects on the viabilities of three kinds of cells; therefore, the optimal CP or CPS concentrations and reaction time were selected for improving viabilities of different cell types. It was also indicated that CP was more effective than CPS for increasing cell viabilities, maybe due to the interaction between primary cells and microbes.

The previous researches have indicated that lactic acid bacteria can synthesize a wide variety of polysaccharides during their growth process (Round et al. 2011; Poole et al. 2018). These polysaccharides can be classified into two kinds, one kind can be tightly linked to the cell surface forming the capsular polysaccharides, which are loosely attached to the extracellular surface, or secreted to the environment as exopolysaccharides (Castro-Bravo et al. 2018). Capsular polysaccharide adhesion to intestinal epithelial cells is believed to help probiotic bacteria to transiently colonize and persist on epithelial cells for decreasing the colonization of intestinal pathogens (Castro-Bravo et al. 2018). Another kind is called extracellular polysaccharides, which can modulate intestinal immunity and reduce the secretion of proinflammatory cytokines (Laiño et al. 2016). *Enterococcus faecalis* can directly produce extracellular polysaccharide (Rossi et al. 2015), which may be the reason why CP is able to improve cell vitality more than CPS in this study. However, the long-term incubation of CP or CPS was harmful to cells, the reason may be due to the secondary metabolites produced by probiotics to influence cell growth.

*Aspergillus oryzae* can produce many kinds of enzymes such as protease and amylase except for AFB_1_-degradation enzyme, which may affect cell paste and growth. The reason why high ADE concentrations could influence cell viability might be due to the high levels of enzymes existing in ADE to damage cells, so low ADE concentration was selected in this study. It was reported that supplementation of *L. bulgaricus* or *L. rhamnosus* could produce significant protective effect against AFB_1_-induced liver damage and inflammatory response (Chen et al. 2019). Moreover, the addition of compound probiotics and mycotoxin-degradation enzyme could prevent broilers from damages induced by AFB_1_ (Zuo et al. 2013). In this study, four kinds of compound probiotics plus AFB_1_-degradation enzyme additions significantly increased the cell viability induced by AFB_1_, inferring that CPDE or CPSADE could alleviate the toxicology induced by AFB_1_ in three kinds of primary cells.

The previous studies have demonstrated that AFB_1_ exposure can induce inflammation response in different cells and organs (Zhang et al. 2019; Wang et al. 2019; Zhao et al. 2019). Inflammation is a response against infection, illness and injury by the excessive expressions of chemokines and inflammatory cytokines such as TNF-α, IL-6 and IL-8 (Barutta et al. 2015; Guo et al. 2015). TNF-α is a proinflammatory cytokine, which can stimulate various kinds of cells to produce chemokines to cause tissue damage and inflammation response (Shanmugam et al. 2016). It can be speculated that the degree of AFB_1_-induced damage may be decreased by suppressing the overexpression of inflammatory cytokines. In this study, AFB_1_ exposure significantly up-regulated the mRNA abundances of IL-6, IL-8 and TNF-α in the three kinds of primary cells, but CPADE or CPSADE addition significantly down-regulated their mRNA abundances in the intestinal and kidney cells except for TNF-α in liver cells, indicating that probiotic combined with ADE could suppress gene expressions of some pro-inflammatory cytokines such as IL-6 and IL-8 (Weninger and Andrian 2003).

NF-κB is an important nuclear transcription factor and a major regulator for anti-inflammatory. The activated NF-κB plays a vital role in inflammatory response by regulating multiple cytokines (Zhang et al. 2018). In response to the inflammation cytokines, inducible nitric oxide synthase (iNOS) can catalyze the production of NOD which is a potent pro-inflammatory mediator (Surh et al. 2001). NOD1 is an innate immune sensor, which consists of a C-terminal leucine-rich region (LRR), central NOD and N-terminal caspase-activating domain (CARD) (Ma et al. 2020). NOD1 plays an important role in response to pathogen infection to induce activation of intracellular signaling pathway, leading to pro-inflammatory response (Caruso et al. 2014; Robertson et al. 2016). Several studies have showed that TLRs and NODs can participate in production of pro-inflammatory molecules to enhance immune responses (Van-Heel et al. 2005; Fritz et al. 2005). It was reported that NLRs, NOD1 and NOD2 had the similar domain architectures and functions, but had the different CARD domain numbers (Trindade and Chen 2020). It was confirmed that NOD1 and NOD2 could activate the classical NF-κB and MAPK pathways related to cell inflammation and apoptosis (Seger and Wexler 2016).

TLRs play the vital roles in innate immune system. The effects of different mycotoxins on gene expression of TLR2, TLR4 and TLR7 have been reported (Chen et al. 2013). It was reported that 600 μg/kg AFB_1_ in broiler diet could simultaneously down-regulate the expressions of TLR2, TLR4 and TLR7 genes in the intestinal tissues of broilers, and decrease the expressions of cytokines such as IFN-γ and TNF-α to reduce the innate immunity of broilers (Wang et al. 2018). However, another research showed that mixed aflatoxins B and G could up-regulate TLR2 and TLR4 transcripts (Malvandi et al. 2013), corresponding with this study, which may due to the dose-dependent effect of aflatoxins (Peng et al. 2016).

In this study, AFB_1_ exposure could up-regulate NF-κBp65, iNOS, NOD1 and TLR2 mRNA abundances in intestinal, kidney and liver cells to cause to the multiple inflammatory pathway responses, in agreement with the previous report (Yan et al. 2020); however, CPADE or CPADE addition could down-regulate their mRNA abundances except for NOD1 and TNF-α in liver cells, indicating that CPADE or CPADE was able to alleviate cell inflammations and damages induced by AFB_1_ through suppressing the pathway activations of NF-κB, iNOS, NOD1 and TLRs.

It can be concluded that CPADE or CPSADE is able to alleviate AFB_1_-induced cytotoxicity and inflammation of chicken embryo primary intestinal epithelium, liver and kidney cells by down-regulating mRNA abundances of inflammation cytokines through suppressing the activations of NF-κB, iNOS, NOD1 and TLRs signal pathways. These findings provide insights into the future development of strategies for CPADE or CPSADE to protect the primary cells from AFB_1_-induced damages.

## Data Availability

All the data presented in the manuscript will be provided upon request.

## Abbreviations

AFB1: Aflatoxin B1
IL-6: Interleukin 6
IL-8: Interleukin 8
iNOS: Inducible nitric oxide synthase
NF-κBp65: Nuclear factor kappa B p65
TNF-α: Tumor necrosis factor α
NOD1: Nucleotide-binding oligomerization domain containing 1
TLR2: Toll like receptor 2
CP: Compound probiotics
CPS: Cell-free compound probiotic supernatant
ADE: AFB1-degradation enzyme
CPADE: Compound probiotics plus AFB1-degradation enzyme
CPSADE: Cell-free compound probiotics supernatant plus AFB1-degradation enzyme
